# The influence of ultra‐processed foods on gut microbiome and inflammatory markers in schoolchildren from Northeastern Brazil

**DOI:** 10.1002/jpn3.70369

**Published:** 2026-02-04

**Authors:** Cristiane Cosmo Silva‐Luis, Paulo César Trindade da Costa, Vinicius José Baccin Martins, José Luiz de Brito Alves

**Affiliations:** ^1^ Department of Nutrition, Health Sciences Center Federal University of Paraíba João Pessoa Paraíba Brazil; ^2^ Department of Biomedicine Science, Health Sciences Center Federal University of Paraíba João Pessoa Paraíba Brazil

**Keywords:** children, dietary profile, gut microbial community, industrialized diet, inflammation

## Abstract

**Objective:**

This study investigated the relationship between the consumption of ultra‐processed foods (UPF), dietary profile, and inflammation on the intestinal microbiome in children.

**Methods:**

A cross‐sectional study was conducted using data from a community‐based controlled trial involving 82 children aged 7–11 years enrolled in public schools in João Pessoa, Paraíba, Brazil. The gut microbiome was assessed by 16S rRNA gene sequencing. Dietary intake was assessed by a 24‐h food recall and UPF intake was estimated using the NOVA system. Anthropometry, socio‐economic variables, and cytokines (IL‐2, IL‐4, IL‐6, IL‐10, IL‐17a, IFN‐γ, and TNF‐α) were also assessed.

**Results:**

Children in the third tertile (higher consumption of UPF) had a higher intake of calories from UPF (*p* < 0.01), trans‐fatty acids (*p* = 0.01), thiamine (*p* = 0.02), while the intake of protein (*p* = 0.01), and copper (*p* = 0.04) was lower. Children in the third tertile had lower abundance of Ruminococcaceae (*p* = 0.04) and Barnesiellaceae (*p* = 0.02) and higher abundance of the Monoglobaceae and Erysipelotrichaceae (*p* = 0.04). Several bacterial genera showed significant correlations with inflammatory cytokines. *Dorea* and *Subdoligranulum* were associated with IL‐17A and IL‐10; *Agathobacter* with IL‐6, IL‐10, and IFN‐γ; *Faecalibacterium* with IL‐10, IFN‐γ, and TNF‐α; *Fusicatenibacter* and Bifidobacterium with IL‐10; and *Roseburia* with TNF‐α (all *q* < 0.05).

**Conclusions:**

A high UPF intake was associated with a poorer‐quality diet and changes in the composition of the gut microbiome, suggesting potential interactions between diet, microbial communities, and immune responses.

## Abstract (highlights)

**What is Known:**

- High ultra‐processed food (UPF) consumption can promote cardiometabolic disease.
- High UPF consumption may be associated with proinflammatory states.
- High UPF consumption can impair the gut microbiome composition.

**What is New:**

- Ruminococcaceae and Barnesiellaceae families were reduced in the highest tertile of UPF consumption.
- *Faecalibacterium* and *Barnesiella* were increased in children with the highest UPF intake.
- Positive correlations were observed between *Dorea* and *Agathobacter* and IL‐10, and between *Faecalibacterium* and IFN‐γ and TNF‐α.

## Graphical abstract

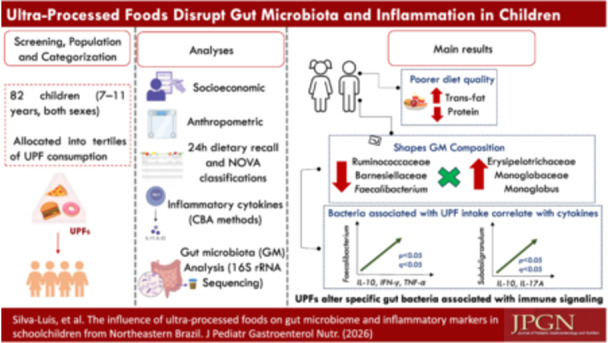

## 1 INTRODUCTION

The gut microbiota plays an essential role in infant health, contributing to energy metabolism, regulating the immune system, maintaining the integrity of the intestinal barrier, and assisting in the digestion of nutrients not processed by human enzymes.^1^ Previous studies have shown that impairment in the composition and diversity of gut microbiota increases the risk of developing chronic noncommunicable diseases.^2^ Diet significantly impacts the gut microbiota, serving as one of the primary factors responsible for influencing its composition.^3^

Humans possess an evolutive capacity to adapt and modify their physiological and behavioral traits in response to environmental influences, particularly during early life stages.^4^ These adaptations can shape traits that remain relatively permanent later in life.^5^ The human gut microbiome is known for its flexibility throughout different stages of life, with research highlighting the early days of life as a vital period for implementing therapeutic dietary strategies.^6^ Nevertheless, there is limited research exploring the relationship between nutritional factors and gut microbiota during childhood.^7^

Ultra‐processed foods (UPFs) consumption has increased markedly in childhood and is associated with cardiometabolic disorders and adverse health outcomes.^8^, ^9^ UPFs are industrial products rich in fats, sugars, emulsifiers, and additives, and poor in fiber, vitamins, and bioactive compounds.^10^ While early‐life exposure to UPFs has been linked to reduced gut microbiota diversity in infancy,^7^ evidence in school‐aged children remains scarce. This developmental period is particularly critical, as high UPF intake may substantially alter gut microbiota composition and function, with potential long‐term health consequences.^2^, ^11^

The gut microbiota plays a central role in balancing pro‐ and anti‐inflammatory responses in the intestine, and diets high in saturated fats, sugar, and salt and low in fiber, along with food additives such as emulsifiers, antimicrobial agents, and artificial sweeteners, can increase gut permeability and inflammation by promoting mucolytic bacteria and endotoxin release.^12^, ^13^ However, the effects of diet and dietary patterns on gut microbiota and inflammation in children remain poorly understood. Therefore, this study investigated the associations between ultra‐processed food consumption, dietary and inflammatory markers, and gut microbiome composition in children from Northeast Brazil.

## 2 METHODS

### 2.1 Ethics statement

The research followed the guidelines established in the Declaration of Helsinki and obtained approval from the Human Research Ethics Committee at the Health Sciences Center of the Federal University of Paraíba, João Pessoa, Brazil (protocol reference number 4.676.103). All procedures adhered to the guidelines outlined in Resolution 466/2012 of the National Health Council. This study is a secondary analysis of a randomized trial conducted in Brazil, registered in the Brazilian Registry of Clinical Trials (ReBEC) under the identifier RBR‐2mzmpxf. Parents received detailed information about the study and provided written consent for the data collection involving their children.

### 2.2 Participants and study design

This cross‐sectional study was conducted in public elementary schools in João Pessoa, Paraíba, Brazil. In 2020, schools serving children aged 7–11 years were identified through the municipal education department's website. After presenting the study to 20 randomly selected schools, 10 agreed to participate, and data were collected from 7 schools across different city regions. Participants were boys and girls aged 7–11 years, classified as obese body mass index‐for‐age (BMI‐for‐age z‐score > +2) or of normal weight (z‐score between ≥–2 and ≤+1). Exclusion criteria included inability to undergo anthropometric assessment, behavioral or psychological disorders, medication use, or medical conditions that could influence the outcomes.

### 2.3 Data collection

The research took place at school between March 2022 and June 2023. All interviews and procedures were conducted by trained dietitians. Parents and children were notified 2 days ahead of time and provided with informational leaflets outlining preparation guidelines for the tests and assessments.

### 2.4 Variables

#### 2.4.1 Dietary assessment and UPF consumption

To assess UPF consumption, two 24 h food recalls (R24h) were obtained—one during a weekday and another on a weekend. The data were organized and analyzed using Brazil Nutri software program, version 1. A Global Diet photo album was employed during interviews to accurately define portion size and reduce potential bias. At least one recall was self‐reported with assistance from mothers or caregivers. The average energy intake (in kilocalories) across the R24h was calculated and portion of total energy from UPFs was expressed as a percentage of the overall energy intake. UPF consumption was evaluated by analyzing tertiles of the percentage of energy derived from UPF, with the first tertile representing the lowest intake and the third the highest. Foods classified as UPFs followed the criteria outlined in the NOVA classification system.^10^

#### 2.4.2 16S ribosomal ribonucleic acid (16S rRNA) sequencing and fecal microbiome analysis

Deoxyribonucleic acid (DNA) extraction from the samples was performed using a MoBio Power Food DNA isolation kit (Mobio Laboratories Inc., USA), following the manufacturer's procedures. The bacteria were identified via high‐throughput sequencing of 16S rRNA V3/V4 region using primers 341F (CCTACGGGRSGCAGCAG) and 806R (GGACTACHVGGGTWTCTAAT). The 16S rRNA libraries were sequenced on the MiSeq Sequencing System (Illumina Inc., USA) using the MiSeq Reagent V2 kit with paired reads of 250 pb (2×250 pb). The KAPA Library Quantification kit for Illumina was used for multiplexing, clustering, and library sequencing reactions (Illumina, USA) on a MiSeq platform.

For the analysis and study of microbial diversity, the quality of the raw data from 16S rRNA V3/V4 was checked using FASTQC. After that, the low‐quality sequences were filtered and trimmed, and the primers were removed using Cutadapt 3.7 software.^14^ The remaining sequences were then processed in DADA2 (1.26) for dereplication chimera removal and clustered into amplicon sequence variants (ASVs).^15^ Subsequently, ASVs were taxonomically identified based on their similarity to sequences in the bacterial database (16S rRNA SILVA 138).^16^ The raw data were submitted to the Sequence Read Archive of the National Center for Biotechnology and identified as PRJNA1356520.

#### 2.4.3 Anthropometry assessment and body composition

Body weight was measured using a digital scale (Omron®, HBF‐514C, São Paulo, Brazil), and height was assessed with a stadiometer (Alturaexata®, Belo Horizonte, Brazil). Nutritional status was determined by body mass index‐for‐age and sex according to World Health Organization standards, using Anthro Plus software (v1.0.4), and classified as obesity (z‐score > +2) or normal weight (−2 ≤ z‐score ≤ +1).

Waist circumference was measured in triplicate with a flexible steel tape (Sanny®, São Paulo, Brazil; 0.1 mm precision), and the mean value was used for analysis. Body fat percentage was estimated from triceps and subscapular skinfold thicknesses, measured in triplicate on the right side using a scientific adipometer (Sanny®; 0.1 mm precision), and calculated according to the Slaughter equation.

#### 2.4.4 Blood samples, biochemical, and cytokines measurements

Serum concentrations cytokines interleukin‐2 (IL‐2), interleukin‐4 (IL‐4), interleukin‐6 (IL‐6), interleukin‐10 (IL‐10), interleukin‐17A (IL‐17A), interferon‐γ (IFN‐γ), and Tumor Necrosis Factor‐α (TNF‐α) were quantified using Th1/Th2/Th17 Cytometric Bead Array (CBA) assay kits provided by Becton Dickinson Biosciences. The concentration of each cytokine (pg/mL) was determined based on the fluorescence intensity of each complex. The analysis utilized an Accuri C6 BD Research (BDR) flow cytometer (San José, CA, USA), with CBA data processed through Flow Cytometric Analysis Program (FCAP) 1.0.1 software.

### 2.5 Statistical analysis

Data normality was assessed using the Kolmogorov–Smirnov test. Results are presented as mean ± standard deviation or median (95% CI). Group comparisons were performed using one‐way analysis of variance with Tukey's post hoc test or Kruskal–Wallis with Dunn's post hoc test, according to data distribution, while categorical variables were analyzed using the chi‐square test. Analyses were conducted in GraphPad Prism (v8.01), with significance set at *p* < 0.05. Gut microbiome sequencing data were processed in R (v4.4.2). Alpha diversity indices (Observed, Chao1, ACE, Shannon, Inverse Simpson, and Fisher) and beta diversity by UPF tertiles were assessed using UniFrac and Bray–Curtis distances. Relative abundance of phyla, families, and genera across UPF tertiles was analyzed using MaAsLin2 and Kruskal–Wallis tests with Dunn's post hoc correction. Spearman's correlation was used to evaluate associations between microbial diversity, bacterial genera, inflammatory markers, and dietary profiles, with correlation strength classified from poor to excellent. Multiple comparisons were adjusted using the Benjamini–Hochberg false discovery rate, with significance defined as *q* < 0.05.

## 3 RESULTS

A total of 82 children were included in the study. The mean age of the children was 9.1 years, 41 boys [50%] and 41 girls [50%]. The characteristics of the study population across tertiles of energy‐percentage‐adjusted UPF consumption are shown in Table 1. Sex, age, nutritional status, and socio‐economic variables were similar among tertiles (Table 1).

**Table 1:** General characteristics of study participants across tertiles of energy‐percentage‐adjusted ultra‐processed food consumption.

	Tertiles of energy‐percentage‐adjusted ultra‐processed food consumption			
Variables	1 (Lowest) *n* = 27	2 *n* = 28	3 (Highest) *n* = 27	*p* value
Anthropometrics				
Sex ‐boy/girl (*n*)†	13/14	15/13	13/14	0.89
Age (years)	9.1 (1.1)	9.1 (1.1)	9.1 (1.4)	0.98
Nutritional status (OB/NW)†	20/7	21/7	20/7	0.99
BMI‐for‐age (z‐score)†	2.2 (1.8‐2.5)	2.4 (2.1‐2.9)	2.4 (2.0‐3.4)	0.44
WC (cm)	71.0 (11.2)	72.1(10.7)	75.9 (14.0)	0.29
BFP (%)	31.1 (9.7)	33.1 (13.0)	35.0 (12.1)	0.48
Family income▲				
Equal to a minimum wage ($232,18), N° (%)†	9 (33.3)	11 (39.2)	8 (29.6)	0.69
Greater than a minimum wage ($232,18), N° (%)†	13 (48.1)	8 (28.5)	9 (33.3)	0.69
Less than a minimum wage ($232,18), N° (%)†	2 (7.4)	5 (17.8)	5 (18.5)	0.69
They did not report their income, N° (%)†	3 (11.1)	4 (14.2)	5 (18.5)	0.69
Bolsa Família federal governamental aid ($114,94)▲				
Yes, N° (%)†	9 (33.3)	13 (46.4)	14 (51.8)	0.36
No, N° (%)†	18 (66.6)	15 (53.5)	13 (48.1)	0.36

*Note*: Values are expressed as mean (SD) or median (95% CI: Lower‐Upper). One‐way ANOVA with Tukey post hoc or Kruskal–Wallis with Dunn's post hoc was used for continuous variables. The Chi‐square test was used for discrete variables grouped into categories.

†Nonparametric data.

▲*Note*: In 2022, the minimum wage of R$1,212 was equivalent to approximately $232.18, and R$600 was equivalent to approximately $114.94, considering the average exchange rate of R$5.22 per dollar.

Abbreviations: %, percent; ANOVA, analysis of variance; BFP, body fat percentage; BIGS, Brazilian Institute of Geography and Statistics; BMI‐for‐age, body mass index for age; CI, confidence interval; cm, centimeter; N°, number; NW, normal weight; OB, obesity; SD, standard deviation; WC, waist circumference.

The dietary profiles of participants by tertiles of UPF consumption are shown in Table 2. Children in the highest tertile of intake consumed more UPF (51.8% vs. 27.2%, *p* < 0.0001), and calories of UPF (181.1 kcal vs. 130.3 kcal, *p* < 0.0001), trans fatty acids (0.4% vs. 0.2%, *p* = 0.01), thiamine (0.46 mg vs. 0.40 mg, *p* = 0.02), and less protein (13.8% vs. 16.5%, *p* = 0.001) than those in the first tertile, and less copper (1.0% vs. 1.2%, *p* = 0.04) than those in the second tertile (Table 2).

**Table 2:** Dietary characteristics of participants across tertiles of energy percentage‐adjusted ultra‐processed food consumption in the diet.

	Tertiles of energy‐percentage‐adjusted ultra‐processed food consumption			
Variables	1 (Lowest) *n* = 27	2 *n* = 28	3 (Highest) *n* = 27	*p* value
Ultra‐processed food intake (%)†	27.2 (25.0–31.6)	39.8 (37.2–41.6)*	51.8 (47.32–55.0) *^#^	<0.0001
Total energy intake of UPF (kcal/d)	561.7 (130.3)	883.1 (227.2)*	1070 (181.1)*^#^	<0.0001
Total energy intake (kcal/d)	1852 (369.3)	2011 (370.3)	2018 (380.9)	0.18
Carbohydrates (%)	53.7 (4.3)	54.3 (5.6)	54.9 (5.0)	0.68
Protein (%)†	16.5 (15.2–17.1)	14.7 (14.1–16.0)	13.8 (12.5–15.2)*	0.0010
Total fat (%)	33.0 (3.6)	32.5 (3.3)	33.1 (3.6)	0.76
Saturated fatty acids (%)†	10.5 (9.9–11.7)	9.9 (9.7–11.0)	10.4 (9.7–11.3)	0.70
Monounsaturated fatty acids (%)	10.0 (1.0)	9.8 (1.1)	9.9 (1.0)	0.77
Polyunsaturated fatty acids (%)†	7.8 (7.5–8.9)	7.9 (7.2–8.5)	8.4 (7.5–9.1)	0.33
Trans fatty acids (%)	0.9 (0.2)	1.1 (0.9)	1.2 (0.4)*	0.01
Cholesterol (mg)†	294.3 (245.7–422.8	300.0 (235.7–328.4)	278.7 (213.5–395.4	0.55
Fiber (g/d)	18.90 (5.3)	20.1 (4.7)	19.0 (4.7)	0.58
Sodium (mg)	2778 (687.1)	3052 (1006)	3074 (567.7)	0.30
Calcium (mg)†	427.2 (336.7–545.0)	468.1 (438.5–561.4)	565.8 (424.5–630.2)	0.05
Magnesium (mg)†	204.3 (183.2–237.0)	229.4 (210.6–253.3)	207.9 (190.5–236.9)	0.13
Phosphorus (mg)	1066 (238.7)	1148 (306.7)	1129 (248.5)	0.49
Potassium (mg)	1782 (438.3)	2009 (519.1)	1802 (448,0)	0.14
Copper (mg)†	1.1 (0.8–1.4)	1.2 (1.0–1.4)	1.0 (0.9–1.2)^#^	0.04
Zinc (mg)	10.0 (2.0)	10.4 (2.9)	9.7 (2.3)	0.52
Iron (mg)†	11.7 (8.3–13.5)	10.9 (9.4–12.6)	11.5 (9.0–12.7)	0.97
Thiamine (mg)	0.99 (0.40)	1.24 (0.40)	1.29 (0.46)*	0.02
Riboflavin (mg)	1.2 (0.56)	1.3 (0.50)	1.3 (0.5)	0.78
Niacin (mg)	14.0 (4.2)	18.4 (6.9)*	16.7 (6.5)	0.02
Pyridoxamine (mg)	0.8 (0.4)	0.8 (0.3)	0.9 (0.3)	0.79
Folate (µg)†	441.1 (334.4–502.6)	432.4 (331.6–489.7)	411.6 (361.2–452.0)	0.87
Cobalamin (µg)†	3.9 (3.3–5.0)	4.3 (3.4–5.3)	3.8 (3.1–4.8)	0.62
Vitamin C (mg)†	111.6 (51.63–164.4)	121.6 (105.2–151.5)	115.6 (102.7–143.9)	0.52
Retinol activity equivalent (RAE) (µg)†	368.5 (265.3–447.3)	528.3 (437.4–657.1)*	425.7 (282.9–636.9)	0.03
Vitamin D (µg)†	1.9 (1.1–2.7)	1.9 (1.4–2.2)	2.0 (1.1–2.4)	0.96
Vitamin E (mg)	6.5 (0.5)	6.7 (0.9)	6.6 (0.6)	0.45

*Note*: Values are expressed as mean (SD) or median (95% CI: Lower‐Upper). One‐way ANOVA with Tukey post hoc or Kruskal–Wallis with Dunn's post hoc was used for continuous variables. The Chi‐square test was used for discrete variables grouped into categories. *p < 0.05 versus tertile 1; #*p* < 0.05 versustertile 2. †Nonparametric data.

Abbreviations: ANOVA, analysis of variance; CI, confidence interval; Kcal, kilocalories per day; mg, milligram; µg, microgram; SD, standard deviation; UPF, ultra‐processed food.

**Figure 1:**
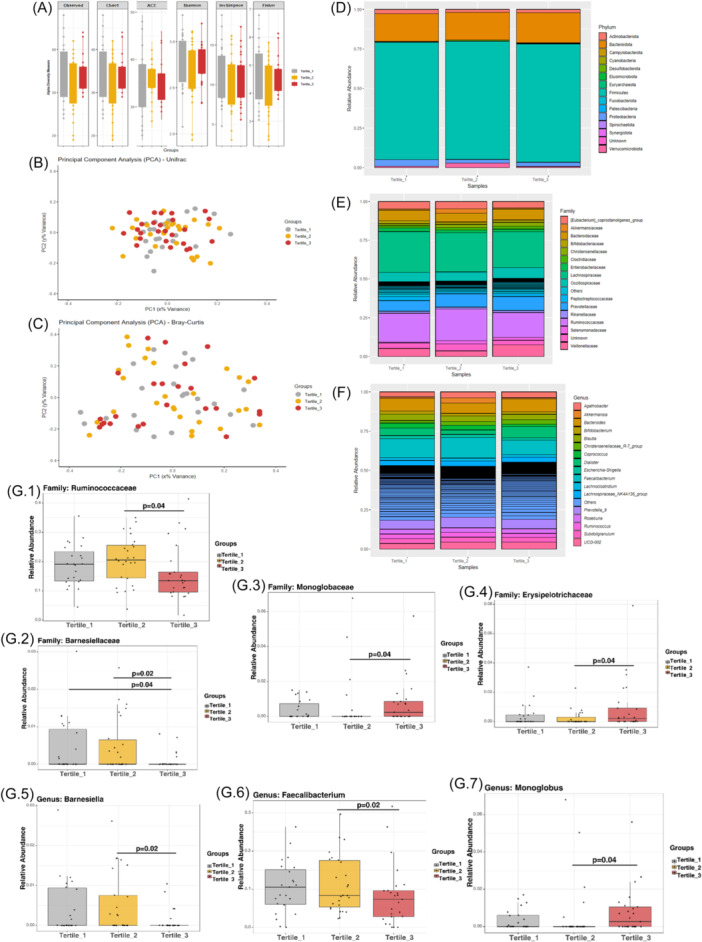
Alpha and beta diversity and relative abundance of fecal bacteria based on 16S rRNA sequencing data of 82 children. Alpha diversity (A), beta diversity weighted Unifrac (B) and Bray‐Curtis (C) distances, phylum level (D) family level (E), genus level (F), abundance of the family Ruminococcaceae (G.1), abundance of the family Barnesiellaceae (G.2), abundance of the family Monoglobaceae (G.3), abundance of the family Erysipelotrichaceae (G.4), abundance of the genera *Barnesiella* (G.5), abundance of the genera *Faecalibacterium* (G.6), abundance of the genera *Monoglobus* (G.7). The data microbiome was expressed as relative abundance and abundance average by tertiles of ultra‐processed food (UPF) and box plot represents median and minimum and maximum by tertiles of UPF consumption.

Alpha and beta diversity indices were similar among groups (Figure 1A–C). The most abundant bacterial phyla were Firmicutes and Bacteroidota, which predominated across all tertiles, followed by Proteobacteria and other less prevalent phyla such as Actinobacteriota and Verrucomicrobiota (Figure 1D). Consistently, the most prevalent bacterial families were Lachnospiraceae, Ruminococcaceae, Prevotellaceae, Bacteroidaceae, Oscillospiraceae, and Veillonellaceae (Figure 1E), while the dominant genera included *Bacteroides*, *Dialister*, *Faecalibacterium*, and *Prevotella_9* (Figure 1F). Children in the highest tertile showed a decrease in the relative abundance of the family Ruminococcaceae (Figure 1G.1) compared to the second tertile (*p* = 0.04, *q* = 0.84) and the family Barnesiellaceae (Figure 1G.2) compared to the second (*p* = 0.02, *q* = 0.84) and first tertiles (*p* = 0.04, *q* = 0.84). In addition, there was a significant increase in the relative abundance of the families Monoglobaceae (Figure 1G.3) and Erysipelotrichaceae (Figure 1G.4) in the third tertile compared to the second tertile (*p* = 0.04, *q* = 0.84). Children in the third tertile had a decrease in the abundance of the genera *Barnesiella* and *Faecalibacterium* (*p* = 0.02, *q* = 0.68, Figure 1G.5 and Figure 1G.6) compared to the second tertile. On the other hand, there was an increase in the relative abundance of the genus *Monoglobus* (*p* = 0.04, *q* = 0.68, Figure 1G.7) compared to the second tertile.

Alpha diversity indices were correlated with the nutrient profile of the diet and with the serum concentration of inflammatory cytokines (Supporting Information: Figure 1A,B). Dietary cholesterol correlated negatively with Simpson (*p* = 0.04, *q* = 0.63), Chao1 and Fisher (*p* = 0.03, *q* = 0.63). Monounsaturated Fatty Acids correlated negatively with Chao1 and Fisher (*p* = 0.04, *q* = 0.63). Positive correlations were observed between inflammatory cytokines and alpha diversity. IFN‐γ correlated positively with Shannon, Simpson and InvSimpson (all *p* = 0.01, *q* = 0.08). IL‐10 correlated positively with Shannon (*p* = 0.003, *q* = 0.05), Simpson and InvSimpson (both *p* = 0.002, *q* = 0.05). IL‐17A correlated positively with Simpson and InvSimpson (both *p* = 0.04, *q* = 0.14). TNF‐α correlated positively with Shannon (*p* = 0.01, *q* = 0.08), Simpson and InvSimpson (both *p* = 0.01, *q* = 0.08) (Supporting Information: Figure 1A,B).

**Figure 2:**
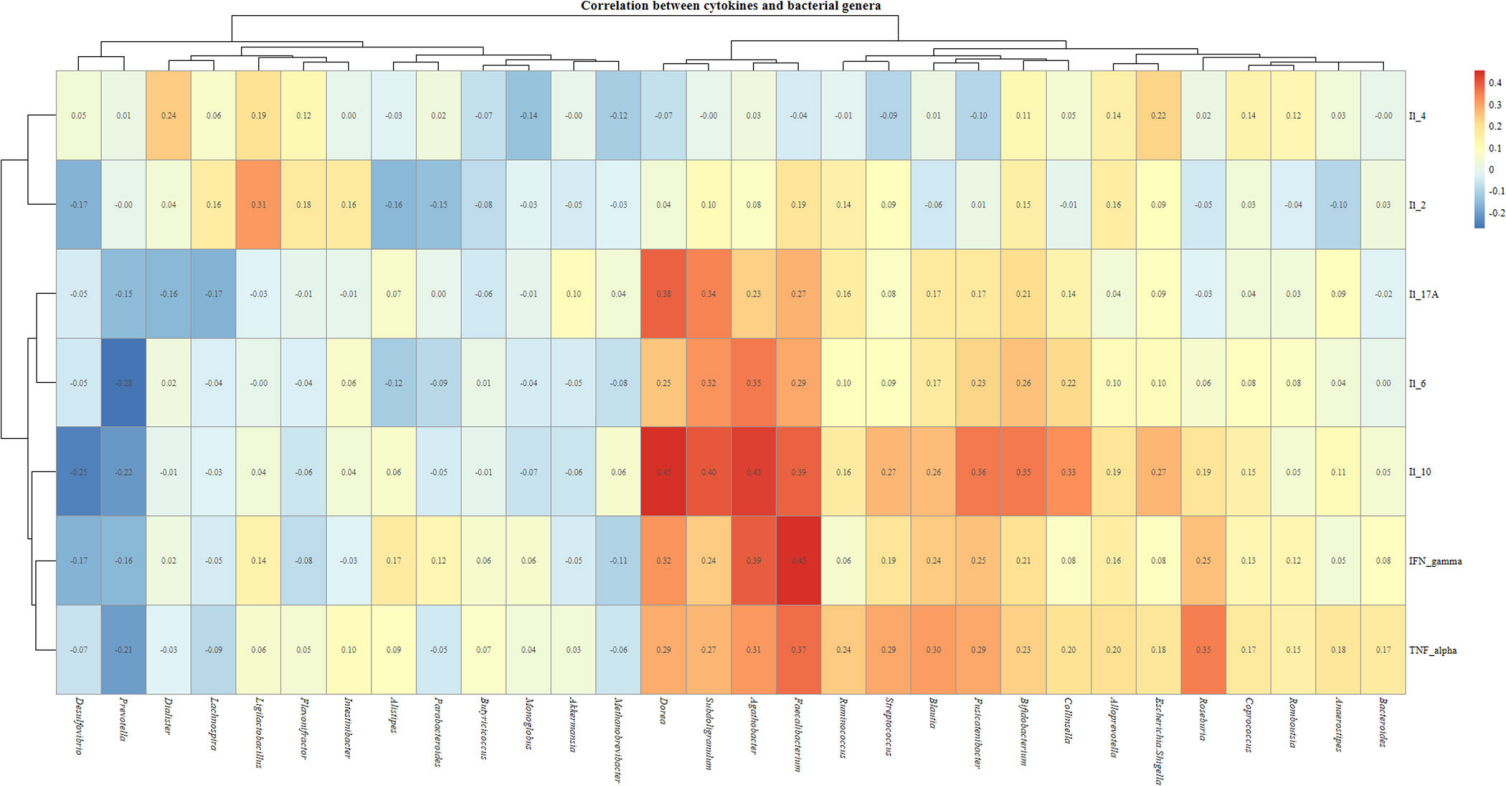
Correlation analysis between the bacterial genera of the 82 children's gut microbiome with serum concentration of inflammatory cytokines. Abbreviations: Interleukin‐17A (IL‐17A); interferon‐γ (IFN‐γ); interleukin‐10 (IL‐10); interleukin‐6 (IL‐6); interleukin‐4 (IL‐4); interleukin‐2 (IL‐2); tumor necrosis factor alpha (TNF‐α).

Multiple bacterial genera showed significant correlations with inflammatory cytokine levels in children (Figure 2). *Dorea* correlated weakly with IL‐17A (*p* = 0.0007, *q* = 0.02) and IL‐10 (*p* < 0.0001, *q* = 0.006), while associations with IL‐6 (*p* = 0.03, *q* = 0.19), IFN‐γ (*p* = 0.005, *q* = 0.07), and TNF‐α (*p* = 0.01, *q* = 0.12) did not. *Subdoligranulum* correlated weakly with IL‐17A (*p* = 0.003, *q* = 0.04) and IL‐10 (*p* = 0.0003, *q* = 0.01). *Agathobacter* showed weak‐to‐moderate correlations with IL‐6 (*p* = 0.001, *q* = 0.03), IL‐10 (*p* < 0.0001, *q* = 0.008), and IFN‐γ (*p* = 0.0005, *q* = 0.02), while correlations with TNF‐α (*p* = 0.007, *q* = 0.08) and IL‐17A (*p* = 0.04, *q* = 0.23) were not sustained. *Faecalibacterium* correlated weakly to moderately with IL‐10 (*p* = 0.0006, *q* = 0.02), IFN‐γ (*p* = 0.002, *q* = 0.006), and TNF‐α (*p* = 0.001, *q* = 0.02), whereas IL‐17A (*p* = 0.01, *q* = 0.14), IL‐6 (*p* = 0.01, *q* = 0.11) did not retain significance. *Fusicatenibacter* showed a weak correlation with IL‐10 (*p* = 0.001, *q* = 0.03), while associations with IFN‐γ (*p* = 0.02, *q* = 0.19) and TNF‐α (*p* = 0.01, *q* = 0.11) were not significant. *Bifidobacterium* correlated weakly with IL‐10 (*p* = 0.002, *q* = 0.03) *Roseburia* exhibited a weak correlation with TNF‐α (*p* = 0.002, *q* = 0.04). These findings suggest potential interactions between gut microbiome composition and systemic immune responses in pediatric populations (Figure 2).

## 4 DISCUSSION

In a previous study of 151 school‐aged children, higher UPF consumption was associated with adverse cardiometabolic and inflammatory markers and poorer diet quality.^8^ Expanding on these findings, the present study showed a higher caloric contribution from UPFs, additional macro‐ and micronutrient imbalances relative to IOM and FAO recommendations, and distinct shifts in gut microbiome composition. Significant correlations between bacterial genera (*Faecalibacterium*, *Agathobacter*, *Dorea*, *Subdoligranulum*, *Fusicatenibacter*, *Roseburia*, and *Bifidobacterium*) and both pro‐ and anti‐inflammatory cytokines suggest potential immunomodulatory pathways linking UPF intake to health outcomes.

Consistent with national and international evidence, UPFs contributed substantially to total energy intake, reaching up to 56% in higher consumption strata and affecting children across socioeconomic groups in Brazil.^17^, ^18^, ^19^, ^20^ In our cohort, children in the highest UPF tertile derived 54.9% of total energy from carbohydrates ( ~ 277 g/day), exceeding the recommended dietary allowance (RDA) but remaining within the acceptable macronutrient distribution range (AMDR) range. Although overall macronutrient intakes were within recommended limits and protein intake exceeded the RDA, high UPF consumption compromised diet quality through excess sugars, unhealthy fats, and low protein quality. Saturated and trans‐fatty acid intakes exceeded recommended thresholds, with higher trans‐fat intake in the highest UPF tertile, while dietary fiber intake remained below the RDA across all groups.

Micronutrient intake showed limited variation by UPF level, except for lower copper and higher thiamine in the highest UPF group, both above the estimated average requirement (EAR). Sodium intake was uniformly high, whereas calcium, potassium, magnesium, and vitamins D and E remained insufficient despite adequate or excessive intakes of phosphorus, zinc, and iron, reinforcing the potential of UPF‐rich diets to undermine nutritional quality during childhood.^18^, ^21^, ^22^, ^23^, ^24^, ^25^

A predominance of the Firmicutes phylum, followed by Bacteroidota, Proteobacteria, Actinobacteriota, and Verrucomicrobiota was found, consistent with the age group and previous studies.^2^, ^26^ Firmicutes and Bacteroidota are the main constituents, with a central role in fiber fermentation and the metabolism of complex polysaccharides, while the other phyla also contribute to the maintenance of intestinal homeostasis.^27^

Lachnospiraceae was the most abundant family in the gut microbiome, followed by Ruminococcaceae and Prevotellaceae. A high proportion of Lachnospiraceae, Ruminococcaceae, and Prevotellaceae was also found in Asian school‐aged children.^28^ On the other hand, a study found a large proportion of Lactobacillaceae in the fecal microbiome of Cambodian children, while Prevotellaceae, Ruminococcaceae and Lachnospiraceae represented the third, fourth, and fifth highest proportions after Enterobacteriacea,^2^ suggesting that the composition of the gut microbiome may be modified by ethnicity. The predominance of Lachnospiraceae, linked to beneficial metabolites and reduced risk of anemia and vitamin A deficiency, but also to obesity,^2^, ^29^ and egg white hypersensitivity,^30^ may reflect the high obesity prevalence in our sample (74.4%). As previously reported, Ruminococcaceae and Prevotellaceae are associated with milk and egg white hypersensitivities. Higher UPF intake was related to reduced relative abundance of Ruminococcaceae and Barnesiellaceae, and increased Erysipelotrichaceae and Monoglobaceae.^30^ Erysipelotrichaceae, implicated in milk and peanut hypersensitivity, obesity, and lipid metabolism,^31^ has been shown to decrease following hypocholesterolemic interventions in animal models.^32^

The most abundant genera identified were *Faecalibacterium*, *Bacteroides*, *Prevotella_9*, and *Dialister*. Children in the highest UPF consumption tertile showed a reduced relative abundance of *Faecalibacterium* and *Barnesiella*, and increased abundance of *Monoglobus*. *Faecalibacterium* has been associated with the absence of iron deficiency anemia,^2^ while *Prevotella*, particularly *P. copri* and *P. melaninogenica*, is linked to anemia in diets high in UPFs and animal‐based foods.^33^ Western diets rich in saturated fat promote Bacteroides, a known lipopolysaccharide (LPS)‐producer involved in innate immune activation,^34^ and variably associated with allergic conditions, including reduced abundance in immunoglobulin E (IgE)‐mediated food allergy,^35^, ^36^ yet increased in cow's milk protein allergy.^37^ *Dialister* is less abundant in children with food sensitization^38^ and has been linked to lower TNF‐α levels.^39^ *Monoglobus*, reduced in milk protein allergy,^40^ has anti‐inflammatory effects through bile acid metabolism, including the production of isoalloLCA and 3‐oxoLCA, molecules that inhibit T helper 17 (Th17) cells and support intestinal homeostasis.^41^, ^42^ Consistent with these immunomodulatory roles, animal models have shown that high‐fat, high‐sugar diets typical of UPFs induce dysbiosis, subclinical intestinal lesions, impaired mucosal immunity, and depletion of *Barnesiella*, a genus protective against colitis.^43^

A large multicenter prospective cohort study of 116,000 adults from multiple countries showed that higher intake of UPF was positively associated with risk of inflammatory bowel disease.^44^ Here, positive correlations were found cytokine IL‐10 and *Dorea*, *Subdoligranulum*, *Agathobacter (*Lachnospiraceae*), Faecalibacterium, Fusicatenibacter*, and *Bifidobacterium* genera. Additionally, a positive correlation was found between *Dorea* and *Subdoligranulum* with IL‐17A. *Agathobacter, Roseburia*, and *Faecalibacterium* genera showed positive correlations with the pro‐inflammatory cytokine IFN‐γ. *Faecalibacterium* also correlated with TNF‐α, and *Agathobacter* correlated with IL‐6. These genera belong to the class Clostridia, except for *Bifidobacterium*, which belongs to the Actinobacteria class. The Clostridia class is known for producing short‐chain fatty acids (SCFA) and immunomodulatory effects, including decreased interleukin‐12 (IL‐12) and IFN‐γ and increased IL‐10.^45^ Despite its association with IL‐10 in this study, *Dorea* has been associated with food allergy sensitization,^38^ highlighting the context‐dependent nature of host‐microbiome interactions. *Agathobacter*, a butyrate producer, contributes to IL‐10 induction and suppression of inflammation^46^ and shows reduced abundance in children with Crohn's disease, as does *Faecalibacterium*, both negatively associated with IL‐2, interleukin‐1 (IL‐1), and IL‐6.^47^ Although, *Faecalibacterium* correlated with IFN‐γ and TNF‐α in the present study, it is widely recognized as a marker of gut health due to its butyrate production and inhibition of the Nuclear Factor kappa‐light‐chain‐enhancer of activated B cells (NF‐κB) pathway.^48^ Butyrate also maintains the integrity of the intestinal barrier and limits LPS translocation.^49^ *F. prausnitzii*, the main species, is notable for its anti‐inflammatory properties, reduced abundance in obesity, and inverse associations with IL‐6 and C‐reactive protein (CRP).^50^

Some limitations should be acknowledged. Recruitment was limited to a subset of schools, potentially reducing sample representativeness and external validity. Dietary records may have introduced underreporting and social desirability bias, particularly for ultra‐processed foods; however, the use of the Global Diet Photographic Atlas and detailed documentation likely mitigated misreporting. Despite these constraints, this study offers an integrated assessment of children's diets by combining nutrient‐ and processing‐based analyses, and the observed associations between ultra‐processed food intake, gut microbiota, and immune biomarkers reinforce the relevance of dietary patterns to pediatric gastrointestinal and metabolic health. The use of tertiles and both relative and absolute intake measures strengthened the robustness and comparability of the findings.

## 5 CONCLUSION

Higher ultra‐processed food consumption was associated with poorer diet quality and distinct alterations in gut microbiome composition in school‐aged children from Northeastern Brazil. Shifts in key bacterial families and genera, together with observed associations between microbial taxa and inflammatory cytokines, suggest interactions between UPF‐rich diets, gut microbial ecology, and immune processes. Larger, longitudinal studies are needed to confirm these findings and to elucidate the underlying mechanisms linking UPF intake to immunometabolic health in childhood.

## CONFLICT OF INTEREST STATEMENT

The authors declare no conflicts of interest.

## Supporting information

Sup_r2.

Supplementary Figure 1. Correlation analysis between the alpha diversity indices (Observed, Chao1, ACE, Shannon, Inv.Simpson and Fisher) with the nutritional profile of the diet and ultra‐processed food consumption (A) and the serum concentration of inflammatory cytokines (B). Data of the 82 children. Abbreviations: Observed richness (Observed); Chao1 richness estimator (Chao1); Abundance‐based Coverage Estimator (ACE); Shannon diversity index (Shannon); Inverse Simpson index (Inv.Simpson); Fisher's alpha diversity index (Fisher); interleukin‐17A (IL‐17A);interferon‐γ (IFN‐γ); interleukin‐10 (IL‐10); interleukin‐6 (IL‐6); interleukin‐4 (IL‐4); interleukin‐2 (IL‐2); tumor necrosis factor alpha (TNF‐α).

## Data Availability

The data that support the findings of this study are available from the corresponding author upon reasonable request. The data are not publicly available due to ethical reasons.
