# Supplementary figures and images for: The influence of ultra‐processed foods on gut microbiome and inflammatory markers in schoolchildren from Northeastern Brazil
Source: J Pediatr Gastroenterol Nutr. 2026 Feb 4;82(4):1140–50. doi: 10.1002/jpn3.70369 (PMC13050821; doi:10.1002/jpn3.70369)

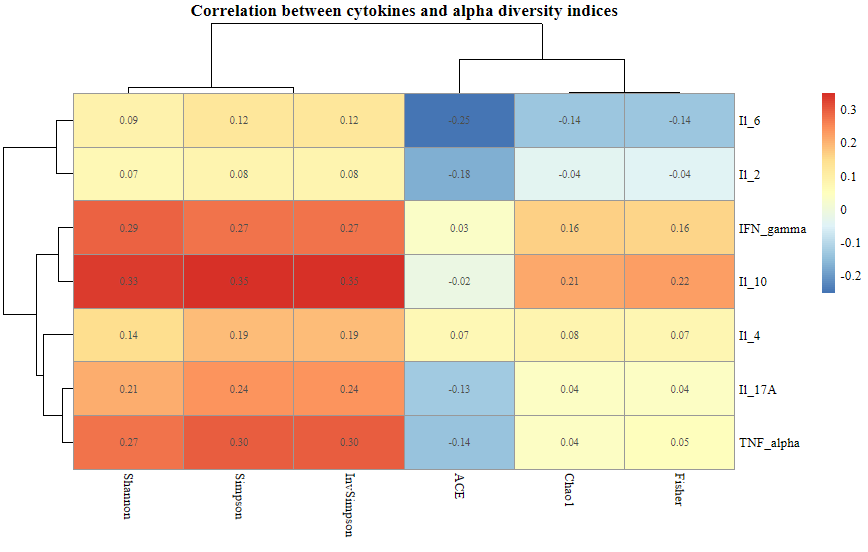

Supplement: Supplementary file 1 — Sup_r2. [file JPN3-82-1140-s001.PNG]

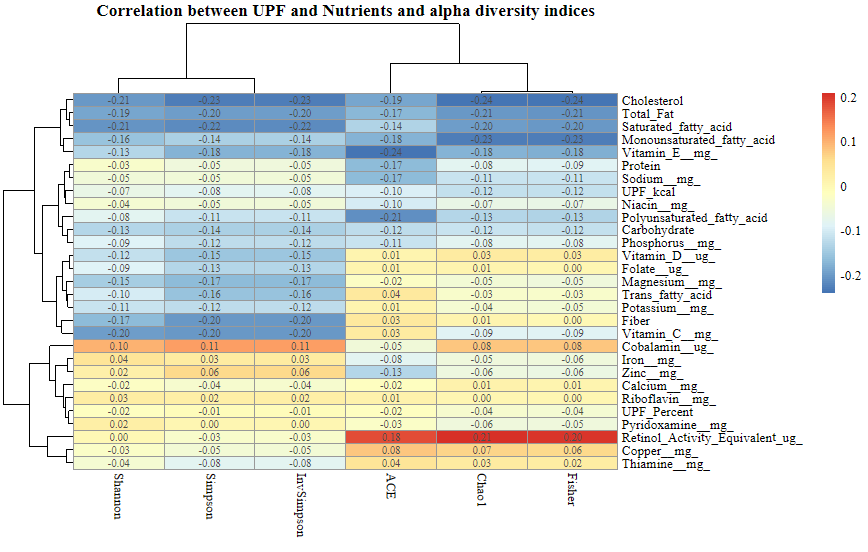

Supplement: Supplementary file 2 — Supplementary Figure 1. Correlation analysis between the alpha diversity indices (Observed, Chao1, ACE, Shannon, Inv.Simpson and Fisher) with the nutritional profile of the diet and ultra‐processed food consumption (A) and the serum concentration of inflammatory cytokines (B). Data of the 82 children. Abbreviations: Observed richness (Observed); Chao1 richness estimator (Chao1); Abundance‐based Coverage Estimator (ACE); Shannon diversity index (Shannon); Inverse Simpson index (Inv.Simpson); Fisher's alpha diversity index (Fisher); interleukin‐17A (IL‐17A);interferon‐γ (IFN‐γ); interleukin‐10 (IL‐10); interleukin‐6 (IL‐6); interleukin‐4 (IL‐4); interleukin‐2 (IL‐2); tumor necrosis factor alpha (TNF‐α). [file JPN3-82-1140-s002.png]
